# Supplementary material for: A dormant overmassive black hole in the early Universe
Source: Nature. 2024 Dec 18;636(8043):594–7. doi: 10.1038/s41586-024-08210-5 (PMC11655357; doi:10.1038/s41586-024-08210-5)
Supplement: Supplementary file 1 — This file contains six figures that show the technical validation steps that were carried out to ensure the robustness of our results, such as a corner plot of the posteriors estimated by our fitting procedure and radial profiles of the galaxy showing the presence of an extended component, or provide additional information, secondary to the core analysis. [file 41586_2024_8210_MOESM1_ESM.pdf]

nature portfolio

https://doi.org/10.1038/s41586-024-08210-5

Supplementary information

A dormant overmassive black hole in the early Universe

In the format provided by the authors and unedited

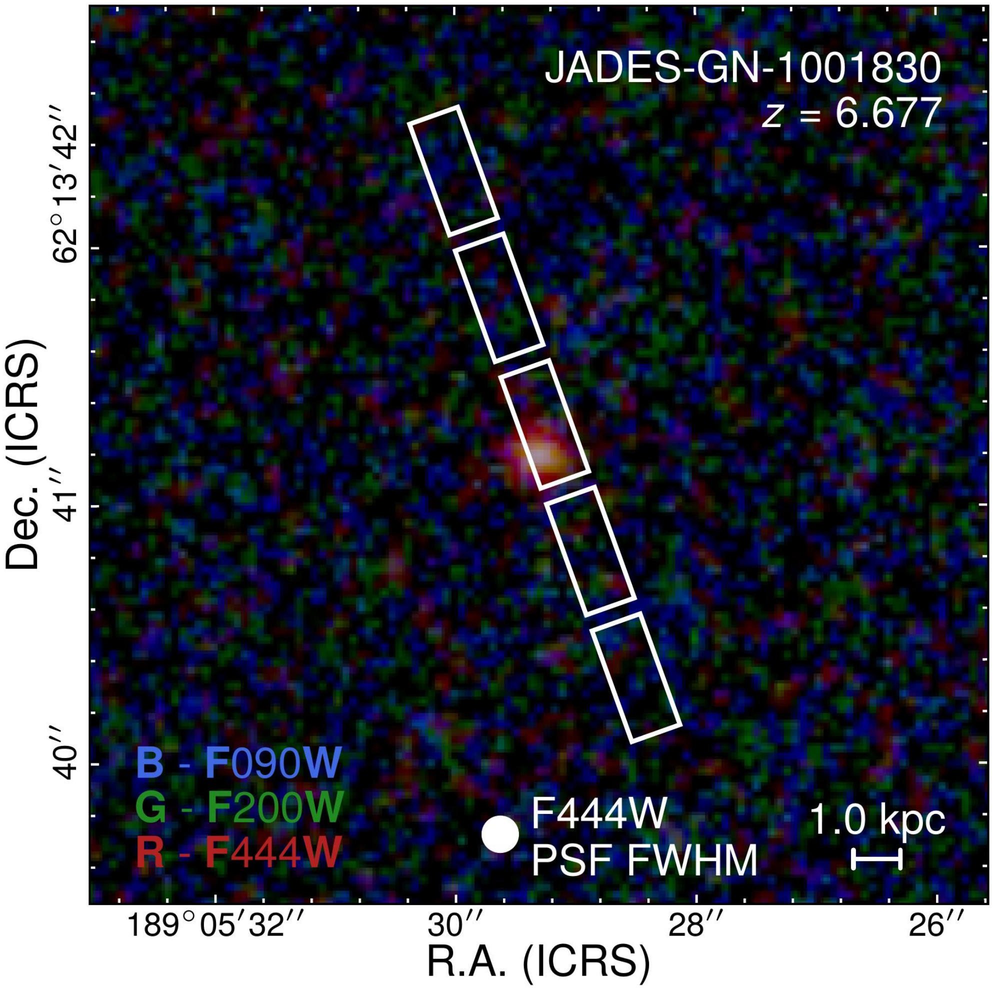

Supplemetray Material Fig. 1: Image of GN-1001830. A red-green-blue (RGB) image of the AGN and galaxy in the F444W, F277W and F115W bands. A 1 kpc physical scale bar is overplotted alongside the FWHM of the PSF in the F444W band. The position of the NIRSpec slit is also overplotted.

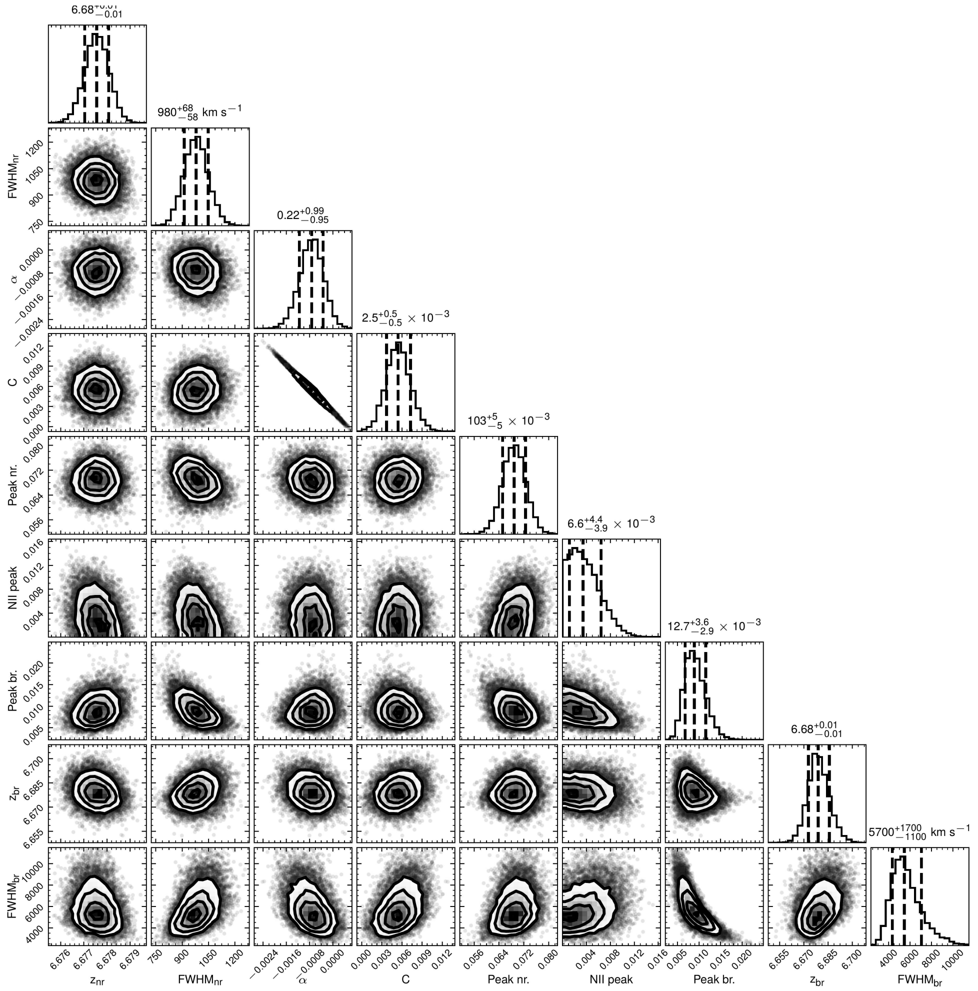

Supplemetray Material Fig. 2: A corner plot summarizing the MCMC fit results of the Hα profile. Here α and C are the continuum slope and normalization respectively, "Peak nr." and "Peak br." show the peak heights of the narrow and broad Hα components respectively. The FWHM values given are not corrected for instrumental broadening. The total spectrum was normalized to unity before fitting, hence peak heights are unitless here. The [S II] doublet parameters are not shown as it is undetected.

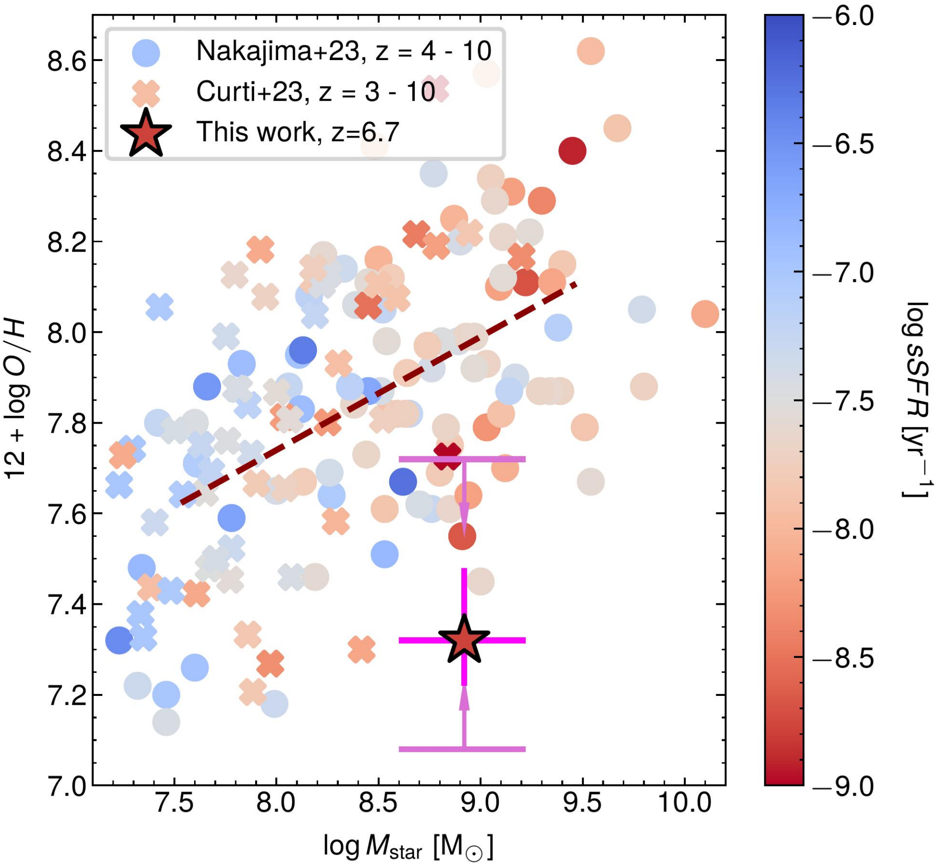

Supplemetray Material Fig. 3: Mass-metallicity relation Location of GN-1001830 (large star with magenta errorbars) relative to the mass-metallicity relation at $z = 4 - 10$ obtained by [1] and [2] (circles and crosses) whose best fit is shown with the brown dashed line. The lighter colored magenta bars show the upper and lower limits for the metallicity of GN-1001830. All markers are colored according to their specific star formation rate, log($sSFR$).

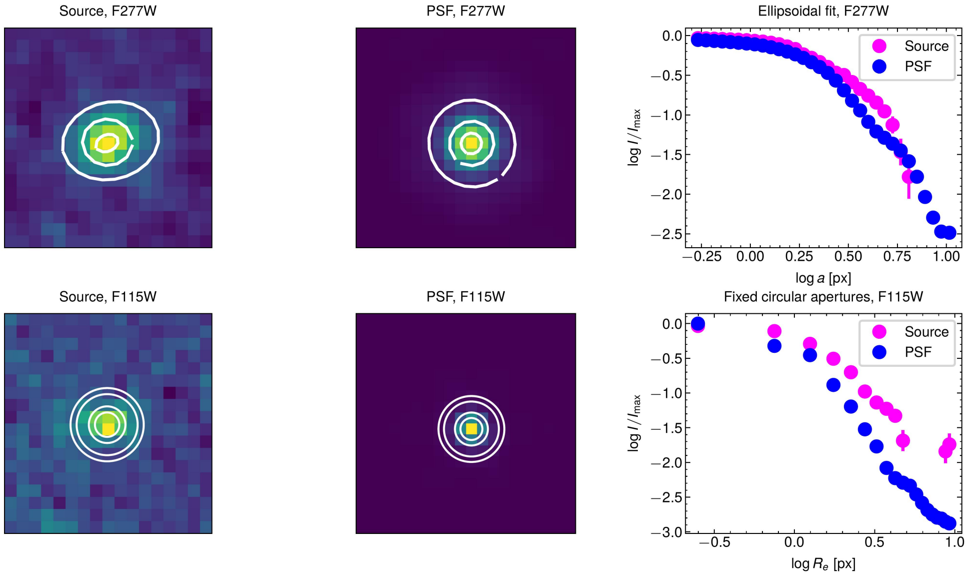

Supplemetray Material Fig. 4: Radial profile diagrams. Comparisons of our object images with the model PSFs in the F277W (top row) and F277W (bottom row) bands. The top row shows the radial profiles for both source (magenta) and PSF (blue) obtained by fitting elliptical isophotes (shown in white in the cutouts). The bottom row shows the radial profiles in F115W band given by measuring fluxes in concentric circular apertures. All radial profiles measured are normalized to the maximum intensity and presented in log-scale. Both methods also reveal GN-1001830 to have a significant extended component.

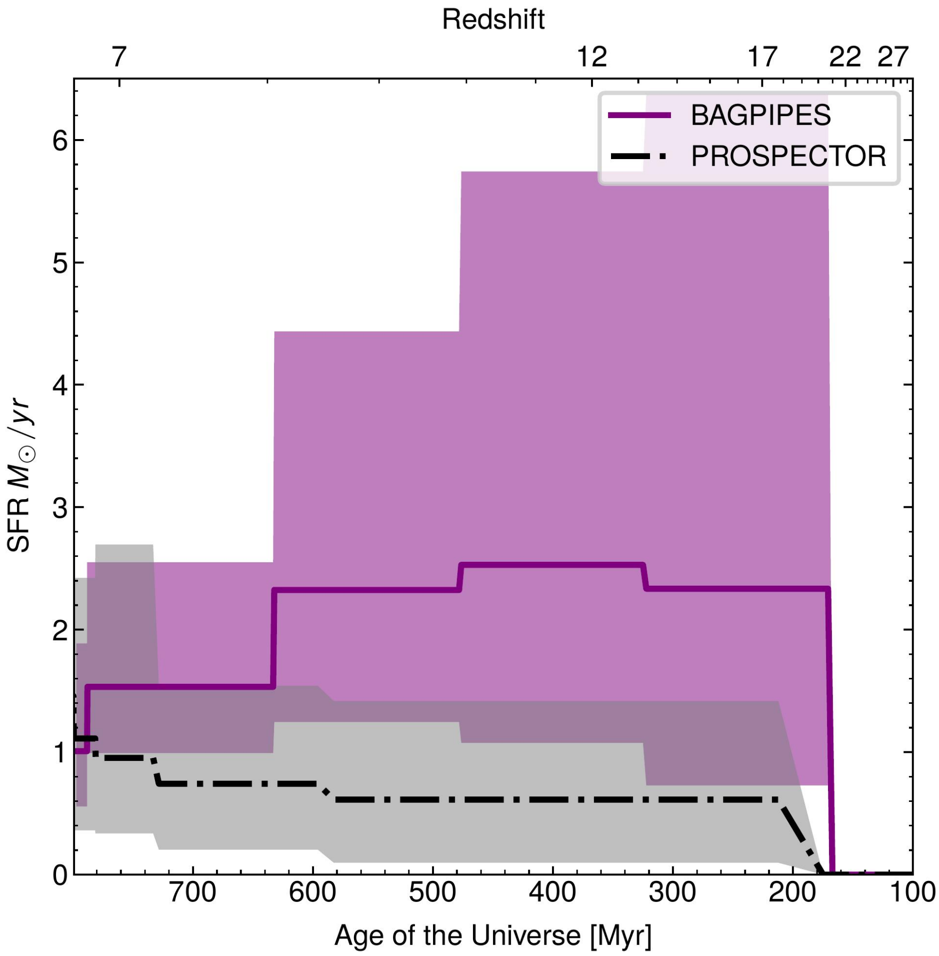

Supplemetray Material Fig. 5: SFH comparisons. A comparison between the SFHs given by the two different codes used. While both SFHs are quite uncertain, they consistently show that the galaxy experienced a roughly constant SFR between 1 and 2 $M_\odot$ yr^{-1} for most of its lifetime.

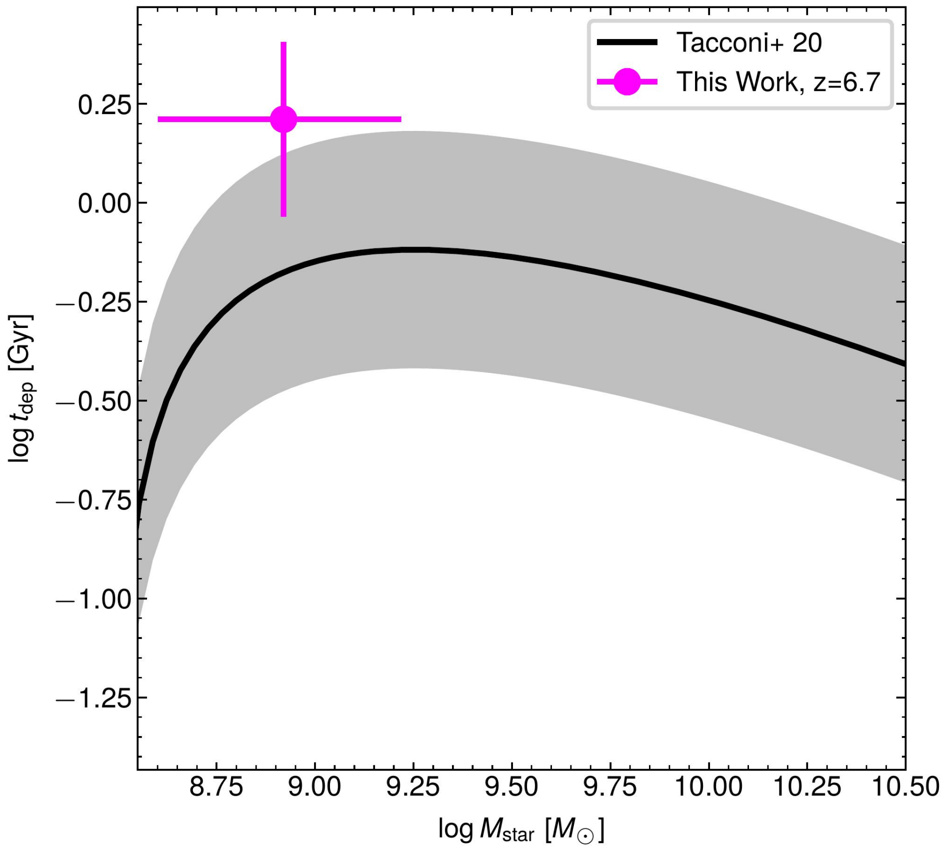

Supplemetray Material Fig. 6: Depletion time. Location of GN-1001830 (magenta point) with respect to the depletion time versus stellar mass scaling relation for sources with the same SFR taken from [3] (solid black line). The grey shading represents the $\sim$0.3 dex intrinsic scatter on the relation.

References

[1] Nakajima, K. *et al.* JWST Census for the Mass-Metallicity Star Formation Relations at z = 4-10 with Self-consistent Flux Calibration and Proper Metallicity Calibrators. *ApJS* **269** (2), 33 (2023). https://doi.org/10.3847/1538-4365/acd556, arXiv:2301.12825 [astro-ph.GA].

[2] Curti, M. *et al.* JADES: Insights into the low-mass end of the mass-metallicity-SFR relation at 3 ¡ z ¡ 10 from deep JWST/NIRSpec spectroscopy. *A&A* **684**, A75 (2024). https://doi.org/10.1051/0004-6361/202346698, arXiv:2304.08516 [astro-ph.GA].

[3] Tacconi, L. J., Genzel, R. & Sternberg, A. The Evolution of the Star-Forming Interstellar Medium Across Cosmic Time. *ARA&A* **58**, 157–203 (2020). https://doi.org/10.1146/annurev-astro-082812-141034, arXiv:2003.06245 [astro-ph.GA].
